# A Novel Crossbeam Structure with Graphene Sensing Element for N/MEMS Mechanical Sensors

**DOI:** 10.3390/nano12122101

**Published:** 2022-06-18

**Authors:** Junqiang Wang, Zehua Zhu, Yue Qi, Mengwei Li

**Affiliations:** 1 National Key Laboratory of Instrumentation Science & Dynamic Measurement, North University of China, Taiyuan 030051, China; zehua_zhu@163.com (Z.Z.); qiyuenuc@163.com (Y.Q.); 2 Academy for Advanced Interdisciplinary Research, North University of China, Taiyuan 030051, China

**Keywords:** graphene, N/MEMS, displacement sensor, pressure sensor

## Abstract

A graphene membrane acts as a highly sensitive element in a nano/micro–electro–mechanical system (N/MEMS) due to its unique physical and chemical properties. Here, a novel crossbeam structure with a graphene varistor protected by Si_3_N_4_ is presented for N/MEMS mechanical sensors. It substantially overcomes the poor reliability of previous sensors with suspended graphene and exhibits excellent mechanoelectrical coupling performance, as graphene is placed on the root of the crossbeam. By performing basic mechanical electrical measurements, a preferable gauge factor of ~1.35 is obtained. The sensitivity of the graphene pressure sensor based on the crossbeam structure chip is 33.13 mV/V/MPa in a wide range of 0~20 MPa. Other static specifications, including hysteresis error, nonlinear error, and repeatability error, are 2.0119%, 3.3622%, and 4.0271%, respectively. We conclude that a crossbeam structure with a graphene sensing element can be an application for the N/MEMS mechanical sensor.

## 1. Introduction

Graphene, a two–dimensional material, has excellent mechanical, thermal, optical, and electrical properties. With its Young’s modulus of up to 1 TPa [1], thermal conductivity reaching 5 × 10^3^ W/m·K [2], light transmittance as high as 97.7% [3], and an ultrahigh carrier mobility of 200,000 cm^2^·V^−1^·s^−1^ [4], it has become the preferred material for the sensing element in various sensors. Moreover, graphene will be suitable for MEMS and NMES mechanical sensors in the future due to two primary factors: the piezoresistive effect because of graphene microstructure changing under external stress and high compatibility between graphene transferring patterning and micro–nano process technology [5,6].

In previous research, graphene sensors have been used to detect multiple different mechanical parameters, such as pressure [7,8], acceleration, and strain [9,10,11,12]. The first typical graphene pressure sensor was proposed by Smith et al. [13,14]. A monolayer graphene membrane was suspended on a cavity in SiO_2_/Si substrate, and a sensitivity of 3.95 μV/V/mmHg was obtained as pressure ranged from 200 to 1000 mbar. There is also an unfavorable phenomenon where liquid remains in the square cavity during the graphene wet transferring process. Subsequently, the electrical property of graphene is inevitably affected, which leads to poor stability of the suspended graphene pressure sensor. A new type of graphene pressure sensor was developed by Zhu et al. [15]. A folded graphene ribbon was placed on the maximum strain region of a suspended square Si_3_N_4_ film, and sensitivity reached up to 8.5 mV/bar with pressure ranging from 0 to 700 mbar. In addition, a more sensitive graphene pressure sensor was developed by Wang et al. [8]. Suspended Si_3_N_4_ for supporting graphene was etched to form substantial numbers of through hole arrays. Owing to the increased strain on the graphene membrane, the pressure sensor showed a sensitivity of 2.8 × 10^−5^ mbar^−1^ under a pressure of 0~400 mbar. Although residual liquid in the cavity can be resolved by the suspended Si_3_N_4_ approach, the measurement range is below 1 MPa due to the process limitations of the suspended Si_3_N_4_ structure. In the past few years, some researchers have also made great progress in graphene based accelerometers. A suspended graphene high–g accelerometer made by Hurst et al. has a high repeatable response to a wide range of 1000~3000 g [16]. Another NMES accelerometer with a suspended double layer graphene ribbon with attached silicon proof mass was fabricated by Fan et al. and could measure an effective acceleration of 20~30 g [10]. These suspended graphene mechanical sensors are simple in structure and exhibit excellent electrical performance. Structural stability and reliability, however, require further improvement. For example, suspended graphene is prone to collapse and rupture [17], and an ultra–thin graphene beam has high processing difficulty and low natural frequency. A crossbeam structure, with its excellent mechanical properties, is commonly used in large impact and high–load environments [18,19]. In addition, the sensitivity of graphene mechanical sensors is related to the effect of stress or strain on the sensing element. Compared with the ordinary ribbon of graphene, the fold pattern can capture multiple responses and improve the detection capability of a sensor.

As the core element of a pressure sensor, the quality of graphene directly affects the piezoresistive performance of the device. Improper operation during the graphene transferring process can easily introduce some defects and residues, such as wrinkling, cracking, and photoresistance [20]. This can cause a risk of Dirac point shift and lead to poor resistivity stability [21,22,23]. Combined annealing for van der Waals force enhancement and water bath heating for cleaning, the performance of graphene can be effectively improved. Another concern is that covering suspended graphene with a passivation layer becomes more difficult. When graphene is exposed to air during fabrication or employment, it is easily contaminated by gas adsorption (e.g., N_2_, O_2_, CO_2_, H_2_O, etc.) or other impurities, causing n–/p–type doping in graphene [24,25]. As a result, graphene resistance sharply changes, sensor reliability shows large decreases, and serving life noticeably shortens [26]. Generally, materials with a strong affinity, such as polymer, h–BN, Al_2_O_3,_ and Si_3_N_4_, are used to protect the graphene sensing element and can improve the electrical properties and long–term stability of sensors [27,28,29,30,31,32]. Compared with Al_2_O_3_, depositing and etching Si_3_N_4_ are relatively convenient methods [33,34], and Si_3_N_4_ processing is also highly compatible with N/MEMS technology, avoiding risk of oxidation under high temperature [35,36,37]. Additionally, it is beneficial for covering Si_3_N_4_, rather than organic polymer or h–BN, on graphene to reduce organic residue pollution in subsequent processing. In this paper, a novel crossbeam structure with a graphene sensing element is presented for N/MEMS mechanical sensors. The core graphene varistors are encapsulated with Si_3_N_4_ film to achieve highly sensitive pressure detection.

## 2. Materials and Methods

### 2.1. Fabrication of N/MEMS Crossbeam Structure

**Figure 1:**
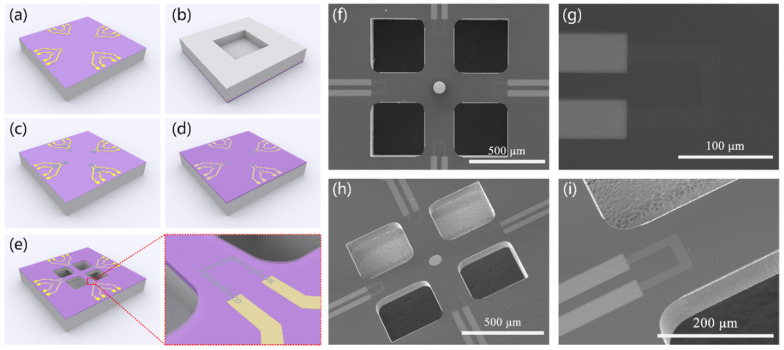
Fabrication process and SEM images of crossbeam structure with graphene sensing element. (**a**) Sputtering Cr/Au electrode. (**b**) Etching deep square cavity on the back of wafer. (**c**) Transferring and patterning monolayer graphene. (**d**) Depositing protective layer of Si_3_N_4_ and etching Si_3_N_4_ to leak out the Pad and sputtering Cr/Au on it. (**e**) Release crossbeam structure. (**f**) Top view of whole chip. (**g**) Top view of single graphene element. (**h**) Tilt observation of whole chip. (**i**) Tilt observation of single graphene element.

The fabrication process is shown in Figure 1. A passivation layer of Si_3_N_4_ with a thickness of 200 nm was first deposited on 2″ Si wafer by plasma enhanced chemical vapor deposition (PECVD). The electrode, 15 nm/25 nm thick Cr/Au, was followed by magnetron sputtering [Figure 1a]. A square 900 × 900 μm cavity was then created on the back of the wafer by deep reactive ion etching (DRIE) [Figure 1b]. Subsequently, the Si wafer was diced into small 17 × 17 mm dies, and the CVD–grown monolayer graphene (TTG, ACS Material, LLC, Pasadena, CA, USA) was transferred and patterned by PMMA assistance and oxygen plasma etching, respectively [Figure 1c]. A thin 150 nm thick Si_3_N_4_ film was also deposited by PECVD to protect the graphene from undesired doping and pollution in the ambient environment. Reactive ion etching (RIE) was used to remove Si_3_N_4_ on the Pad, and Cr/Au with a thickness of 25/100 nm was sputtered on the Pad [Figure 1d]. Finally, four square cavities with dimensions of 350 × 350 μm were etched by DRIE to create a crossbeam with a length of 900 μm, width of 200 μm, and thickness of 40 μm [Figure 1e].

The crossbeam structure with the graphene sensing element was firstly measured using a scanning electron microscope (Quanta 250, FEI, Inc., Hillsboro, OR, USA). Figure 1f,g shows the top view of the whole chip and single graphene element, respectively. Four identical graphene piezoresistors were arranged at the root of crossbeam. Measurement results of the crossbeam, 900 μm in length and 200 μm in width, confirmed the consistency with the design. The size of the folded graphene ribbon was determined as 30 μm long and 10 μm wide. As shown in Figure 1h,i, the crossbeam structure was also inspected using a SEM with a tilt angle. It is clear that the crossbeam thickness at the root was thicker than that in the middle because of the universal feature of the DRIE process. Nevertheless, it had almost no influence on the mechanical properties of crossbeam.

### 2.2. Schematic of Transfer Process of Graphene Layer

**Figure 2:**
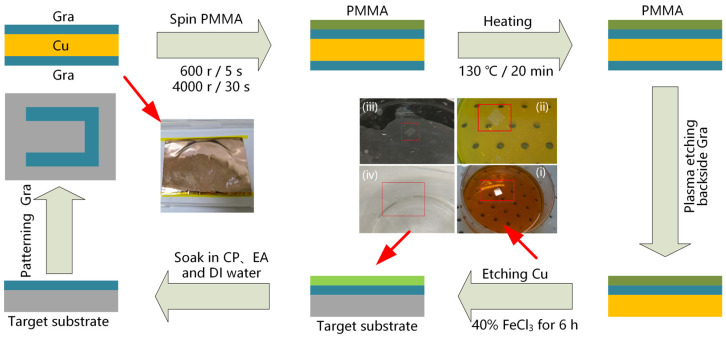
Schematic of transferring graphene layer to target substrate (including patterning graphene).

Single–layer graphene (Gra) was grown on a Cu substrate. After being cropped into a 1.5 × 1.5 cm sample, we coated the graphene on Cu foil with polymethyl methacrylate (PMMA) using a spin coater at speeds of 600 rpm/5 s and 4000 rpm/30 s. The PMMA/Gra/Cu/Gra sample was placed on a hot plate at 130 °C for 20 min. Then, the backside graphene of the sample was etched by O_2_ plasma, and the Cu substrate of supporting Gra was etched with 40% FeCl_3_ solution for about 6 h. After, the PMMA/Gra sample was transferred to the target substrate and heated on a hot plate at 85 °C for 30 min. The PMMA layer was dissolved in acetone (CP) solution at 50 °C for 10 min, and the target was cleaned with alcohol (EA) solution and deionized (DI) water. Finally, photolithography technology and O_2_ plasma etching were used for patterning graphene. The parameters of the O_2_ plasma etching were as follows: the power was 60 W, gas flow rate was 30 sccm, and etching time was 3 min. The schematic of transferring the graphene layer to the target substrate is shown in Figure 2.

## 3. Results and Discussion

### 3.1. Physical and Electrical Characteristics of the N/MEMS Graphene Units

**Figure 3:**
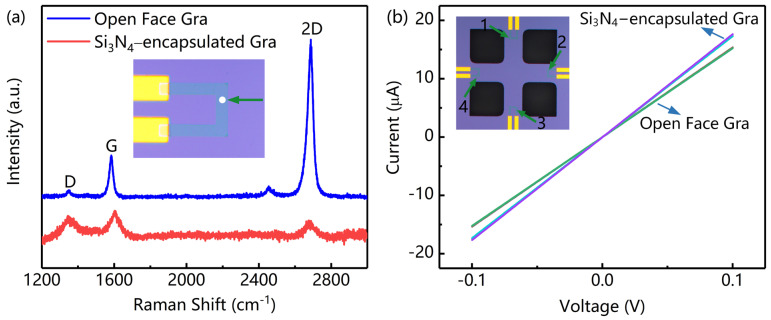
Physical and electrical characteristics of graphene sensing element. (**a**) Raman spectra of graphene sensing element before and after depositing Si_3_N_4_. (**b**) I−V curve of graphene sensing element with open face and Si_3_N_4_ protective layer.

Raman spectra (HR–800, Horiba Scientific, Inc., Paris, France) were used to analyze the stacking and defects of graphene before and after depositing Si_3_N_4_. As shown in Figure 3a, the characteristic G and 2D peaks, as well as a weak D peak (defect-related), originating from the CVD–grown monolayer graphene were clearly visible. After depositing the Si_3_N_4_–protected layer, the intensity of the D peak rapidly increased; meanwhile, the intensity ratio of the 2D peak to the G peak (I_2D_/I_G_) decreased immensely from 3.08 to 0.60. Moreover, a slight shift in the G and 2D peaks was also observed [38,39]. The main explanation for the above phenomena is that the introduction of external atoms disrupts the lattice structure of graphene. Current–voltage (I–V) characteristics were precisely demonstrated by a probing station united with a semiconductor parameter analyzer (B1500A, Keysight, Inc., Santa Rosa, CA, USA). The measurement results are shown in Figure 3b. Compared with the resistance of the open face graphene sensing element from 497.5 to 502.4 Ω·sq^−1^, Si_3_N_4_−encapsulated graphene resistance ranged from 566.1 to 570.8 Ω·sq^−1^. This indicated that graphene can retain high quality and consistency after overlaying a Si_3_N_4_ protective layer.

**Figure 4:**
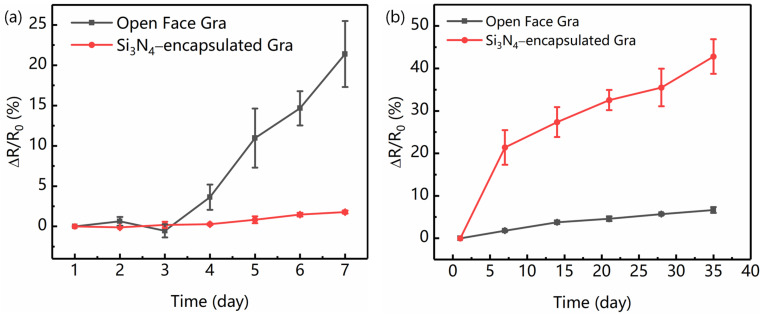
Stability of graphene sensing element with and without the top Si_3_N_4_ layer: (**a**) 7 and (**b**) 35 days later.

To further investigate the effect of a Si_3_N_4_ layer on the stability of the graphene sensing element, samples with and without the top Si_3_N_4_ layer were placed in ambient air (20~25 °C and relative humidity 40~60%). From Figure 4a, after 7 days, the graphene resistance without top Si_3_N_4_ increased by ∆*R*/*R*_0_ = 25.8%, where *R*_0_ is the initial resistance. In contrast, the relative resistance protected with a Si_3_N_4_ layer was almost unchanged, showing higher stability. The main reason is that Si_3_N_4_ can effectively isolate graphene and avoid water or air doping [38]. After 35 days, the unprotected graphene resistance continued to increase, while the protected one remained only slightly increased, as shown in Figure 4b. The corresponding changes in resistance were 7.4% and 46.3%, respectively. We confirmed that the stable performance of graphene resistance is well preserved due to the Si_3_N_4_ protecting graphene from serious environmental pollution or doping [36].

### 3.2. Mechanical and Electrical Characteristics of Displacement Sensor

**Figure 5:**
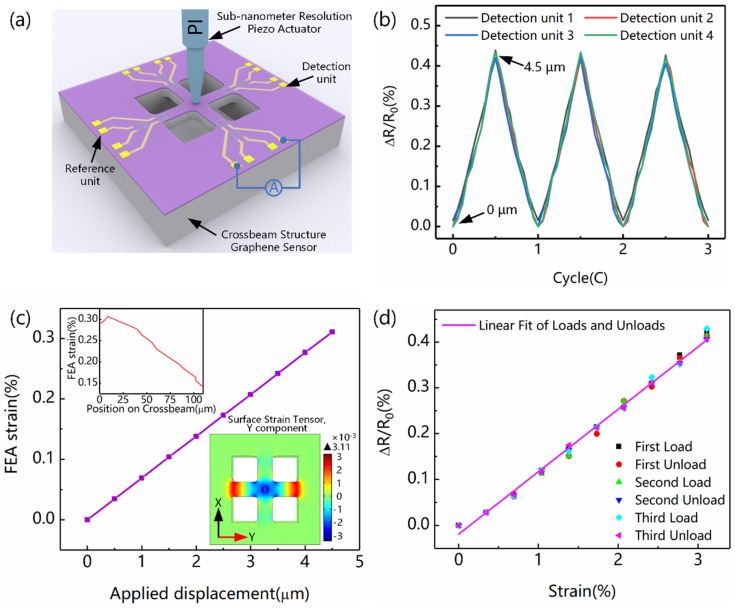
Experimental characterization of crossbeam structure with graphene sensing element. (**a**) Schematic of experimental setup. (**b**) Measurement results of displacement resistance. (**c**) Strain–displacement diagram of crossbeam root by FEA. (**d**) Strain–resistance results of the detection unit 1.

Tests of the piezoresistive effect for the graphene sensing element on the crossbeam structure were then performed in an uncomplicated experiment. The setup schematic is shown in Figure 5a. There were eight independent sensing units of the same size, distributed on the surface of chip. Four of them were arranged outside the crossbeam as references and did not sense mechanical signals such as strain, on which electrical tests were mainly performed during fabricating to judge the compatibility and reliability of the process. Other graphene sensing units were located at the root of the crossbeam and were utilized to detect the beam strain signal. A piezo actuator with subnanometer resolution (P–841, Physik Instrumente, Inc., Karlsruhe, Germany) was then used to compress the center of the crossbeam. As the actuator simulated external force to produce a tiny displacement, the bend deformation of the crossbeam correspondingly occurred; meanwhile, the graphene sensing element was affected by stress and strain. The resistances of the four graphene varistors were recorded through a digital multimeter (34461A, Keysight Technologies, Inc., Santa Rosa, CA, USA). Measurement results are shown in Figure 5b. After three cycles of the load–unload experiment, with displacement ranging from 0 to 4.5 μm, the changes in resistance and displacement exhibited an apparent positive linear correlation. Moreover, the output results of the four detection units had high consistency, which further verified that the strain generated at the root of the crossbeam remained the same as when the pressure was applied at the center. The piezoresistive effect of graphene is independent of random crystal orientation and multigrain graphene flake [13].

The piezoresistive effect of the graphene sensing element, through the strain−induced resistance change, was further investigated by finite element analysis (FEA). During FEA simulation, differential displacement load, ranged from 0 to 4.5 μm with a step of 0.5 μm, was applied on the center of the crossbeam structure. Although the maximum displacement was located at the center of the crossbeam, the maximum stress (i.e., strain) appeared at the root of the crossbeam. As shown in Figure 5c, the typical surface maximum strain distribution at the root of a single beam was extracted under different displacements. The internal illustrations showed the strain distribution within the range of 110 μm at the root and the Y component of the surface strain tensor under the applied displacement of 4.5 μm. The X component of the tensor was the same as that of the Y component, and they were perpendicular to one another. We found that the effective strain region was located within 110 μm of the root of the crossbeam. In our work, the graphene sensing element was arranged within 15 μm, which included the position of maximum strain. Because strain was the main factor causing the piezoresistive effect of graphene, the maximum strain parameter was used instead of the displacement parameter. Because the output characteristics of the four detection units were basically similar, only one of them was analyzed in detail. The corresponding result of strain–resistance is shown in Figure 5d. The gauge factor (G) of the graphene piezoresistor was defined as the rate of resistance change to strain (ΔR/R_0_/ε). Finally, G = 1.35 was obtained in this work, which is similar to the G = 1.6 for CVD graphene obtained in previous research [15]. This also demonstrates the feasibility of arrangement of the graphene sensing element.

### 3.3. Mechanical and Electrical Characteristics of Pressure Sensor

**Figure 6:**
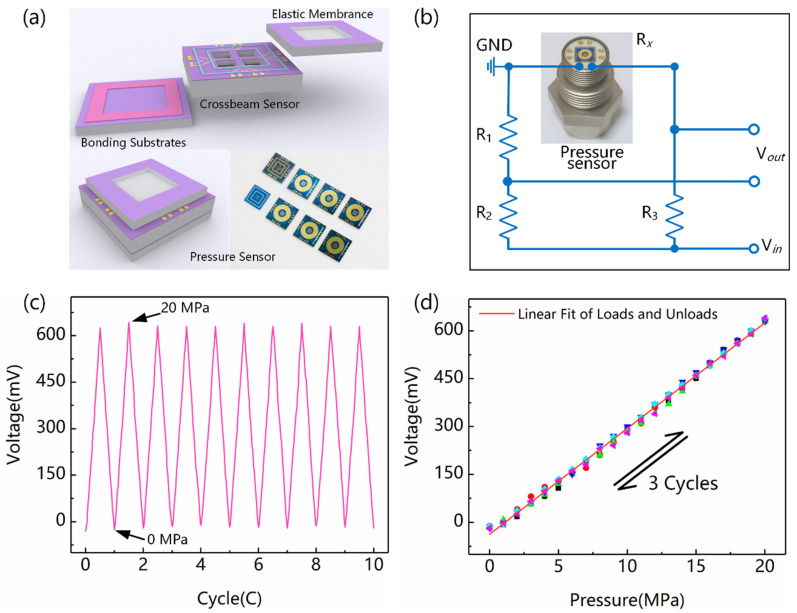
Packaging and testing of graphene pressure sensor based on crossbeam. (**a**) Packaging schematic and actual chip of sensor. (**b**) Shell assembling and electrical connection of sensor. (**c**) Test results of 10 load–unload cycles in full–range condition. (**d**) Test results of 3 reciprocating cycles.

A new type of graphene pressure sensor was developed by stacking an elastic diaphragm, crossbeam chip, and substrate through bonding technology. Figure 6a shows a schematic diagram of the pressure sensor packaging and actual sensor chip. Furthermore, the chip was assembled on the shell, and the signal was led out by wire bonding. In addition, a wheatstone bridge with a constant current supply was used to detect the electrical conductivity changes in a graphene nanofilm caused by external pressure, as shown in Figure 6b. The pressure sensor was then tested by using a self–made oil pressure calibration machine. Figure 6c shows the voltage output results of 10 cycles in the range of 0~20 MPa, with an interval of 1MPa. The graphene sensing element was not only directly covered by Si_3_N_4_, but also isolated from the environment by multistack bonding. Double protection for graphene can greatly improve the repeatability and stability of a sensor. The results of three reciprocating cycles were extracted, as shown in Figure 6d. The calculated sensitivity of the sensor was 33.13 mV/V/MPa. Correspondingly, the hysteresis error, nonlinear error, and repeatability error reached 2.0119%, 3.3622%, and 4.0271%, respectively.

## 4. Conclusions

In conclusion, a novel crossbeam structure was employed for graphene N/MEMS mechanical sensors in this work, which significantly overcomes some disadvantages in the performance stability and fabrication process of a suspended structure. Defect free structure and high–quality graphene was demonstrated by a SEM inspection, Raman analysis, and I–V measurement. The results of stability tests further confirmed that Si_3_N_4_ protection can prolong the working life of graphene devices. The piezoresistive effect of the graphene sensing element was gradually confirmed through displacement resistance measurements and strain–resistance analysis. In the end, a gauge factor of 1.35, near to that of CVD graphene, was reached. Based on the crossbeam structure chip, the sensitivity of the graphene pressure sensor was as high as 33.13 mV/V/MPa under a wide range of conditions. Other static specifications also demonstrated high repeatability and reliability. This indicates that the crossbeam structure is an extremely useful application for graphene N/MEMS mechanical sensors.

## Data Availability

Not applicable.
